# An industry consensus study on an HPLC fluorescence method for the determination of (±)-catechin and (±)-epicatechin in cocoa and chocolate products

**DOI:** 10.1186/1752-153X-5-39

**Published:** 2011-07-05

**Authors:** Laura Shumow, Alison Bodor

**Affiliations:** 1 National Confectioners Association, 1101 30th St NW, Suite #200, Washington, DC 20007, USA

## Abstract

**Background:**

This manuscript describes the results of an HPLC study for the determination of the flavan-3-ol monomers, (±)-catechin and (±)-epicatechin, in cocoa and plain dark and milk chocolate products. The study was performed under the auspices of the National Confectioners Association (NCA) and involved the analysis of a series of samples by laboratories of five member companies using a common method.

**Methodology:**

The method reported in this paper uses reversed phase HPLC with fluorescence detection to analyze (±)-epicatechin and (±)-catechin extracted with an acidic solvent from defatted cocoa and chocolate. In addition to a variety of cocoa and chocolate products, the sample set included a blind duplicate used to assess method reproducibility. All data were subjected to statistical analysis with outliers eliminated from the data set.

**Results:**

The percent coefficient of variation (%CV) of the sample set ranged from approximately 7 to 15%.

**Conclusions:**

Further experimental details are described in the body of the manuscript and the results indicate the method is suitable for the determination of (±)-catechin and (±)-epicatechin in cocoa and chocolate products and represents the first collaborative study of this HPLC method for these compounds in these matrices.

## Background

The use of cocoa has been documented for almost 4,000 years. The first population thought to consume the material was the Mesoamericans [1,2]. In the past decade there has been increasing interest and numerous publications on the putative health effects associated with the moderate consumption of cocoa and chocolate products [3-5]. In a parallel fashion, several groups initiated studies into the potential agents responsible for cardiovascular effects with the flavanols, (±)-catechin and (±)-epicatechin being candidate compounds [6].

The growing interest in these compounds resulted in a plethora of methods for quantification in various foodstuffs including tea, wine, grapes and chocolate. While other analytical methods have been used, HPLC was the predominant method developed [7-13]. A thorough literature search using Google and Pubmed resulted in thousands of citations on polyphenol analysis and almost 900 citations on flavanol analysis by HPLC in chocolate, indicating the recent explosive growth in methods for these analytes. Considering the increased interest in the cocoa flavanols' potential cardiovascular effects, a standard quantification method would be pertinent for accurate determination of dose-response effects in clinical trials. There is both an ISO and an Institute for Nutraceutical Advancement (INA) method for flavanols in tea, but not yet a standard method for flavanol quantification in chocolate and cocoa [14,15].

With this as background, the National Confectioners Association (NCA) convened an analytical chemistry working group to develop a consensus HPLC method. This group conducted a collaborative study using samples provided by NCA to establish a method to quantify (±)-catechin and (±)-epicatechin in cocoa and chocolate and make it available to the industry.

## Experimental

### Scope and Applicability

This method is applicable for the analysis of (±)-epicatechin and (±)-catechin in cocoa powder, chocolate liquor and formulated chocolate products. This ring trial only included pure chocolate; any products containing inclusions (such as fruit or nuts) may not be appropriate for this method due to potential interference.

#### A. *Principle*

This method determines the (±)-catechin and (±)-epicatechin content of cocoa and chocolate products. Fat is removed from the sample in order to prevent potential interference and protect the column by using multiple hexane extractions. Defatted samples are then dried for subsequent extraction of analytes. Defatted, dried samples are extracted, with sonication, at 40°C for 15 minutes using an acetone: water: acetic acid (70: 29.5: 0.5) solvent mixture. Extracted samples are then centrifuged to remove insoluble materials and brought up to a defined volume. The extracts are filtered into HPLC vials for chromatographic analysis. (±)-Catechin and (±)-epicatechin are separated by a reverse phase mechanism on a C18 column with an acidic acetonitrile-water mobile phase gradient. Analytes are detected and quantified by their fluorescence, with excitation at 280 nm and emission at 315 nm.

#### B. *Apparatus*

**(a) ***HPLC system: *With solvent degasser, binary gradient pumping, gradient mixer, injector capable of 10 μL injection (either autosampler or manual), column oven, fluorescence detector and data analysis system

**(b) ***Chromatography column: *Reversed phase HPLC column octadecylsilane (ODS; C18) derivatized silica reversed phase HPLC column, pore size from 100 - 125 A, are recommended. Recommended column: *Phenomenex Luna, 5 μm, C18(2), 100A, 250 × 3.0 mm *(alternate columns may be used if they provide acceptable resolution)

**(c) ***Analytical balance: *Readability 0.1 mg or lower

**(d) ***Pipettes: *Capable of accurately delivering 20-1000 μL; 1-5 mL

**(e) ***Vials: *2 mL, amber glass, screw cap, for storing Stock Standard solutions and for holding filtered HPLC sample prior to injection

**(f) ***Test Tubes: *Screw capped, with caps, capable of holding at least 10 mL

**(g) ***Volumetric flasks: *10 mL, 20 mL, 50 mL and 100 mL, Class A, glass

**(h) ***Centrifuge tubes: *Plastic, for single use, 50 mL, screw cap (air tight)

**(i) ***Vortex Mixer*

**(j) ***Ultrasonic Bath*

**(k) ***Flame-Proof Centrifuge: *For centrifuging 50 mL tubes at 2500 × *g*

**(l) ***Syringe filters: *For filtering HPLC samples, 0.45 μm PVDF, PTFE or hydrophilic polypropylene, 13 or 25 mm diameter (Nylon filters are not recommended due to potential adsorption of metabolites)

**(m) ***Syringe: *All plastic, 1 mL to 5 mL as appropriate

**(n) ***Glass beads: *(approx. diameter 5 mm)

#### C. *Reagents*

**(a) ***Water: *High purity deionized water, filtered through a 0.45 μm or smaller pore filter

**(b) ***Acetonitrile: *HPLC grade

**(c) ***Hexane: *HPLC grade

**(d) ***Acetone: *HPLC grade

**(e) ***Acetic Acid: *Glacial

**(f) ***Extraction Solvent: *Mix 700 mL Acetone, 295 mL Water and 5 mL Acetic Acid

**(g) ***Mobile Phase A: *0.2% Acetic Acid in Water. Add 2 mL Acetic Acid to 1 L Water.

**(h) ***Mobile Phase B: *0.2% Acetic Acid in Acetonitrile. Add 2 mL Acetic Acid to 1 L Acetonitrile.

**(i) ***Standards: *(±)-Catechin hydrate, purity ≥ 98%, Sigma-Aldrich C1251-5G or equivalent; (±)-epicatechin, purity ≥ 98%, Sigma-Aldrich E4018-1G or equivalent. Certificate of analysis from supplier is required for purity correction of each new lot number.

#### D. *Standards and Reagent Blank Preparation*

**(a) **(i.) *Stock standard solution A (approx 1000 μg/mL): *Into a 50 mL volumetric flask, accurately weigh approximately 50 mg (±)-catechin hydrate and 50 mg (±)-epicatechin and record the weights. Add extraction solvent and mix or sonicate to dissolve. Bring to 50.00 mL with extraction solvent and mix. Label as Stock standard A.

(ii.) *Stock standard solution B (approx 100 μg/mL): *Pipette, using a Class A volumetric pipette, 5 mL of Stock standard A into a 50 mL volumetric flask and dilute to volume with extraction solvent. Label as Stock standard B.

**(b) ***Obtain loss on drying (important for (±)-catechin hydrate)*: Crystal water is not stoichiometrically distributed; for (±)-epicatechin loss on drying normally equals 0) and HPLC purity of analyte from supplier's certificate of analysis for each new lot number to calculate purity: Purity (%) = [100 (%) - loss on drying (%)] * HPLC purity (%)/100 (%)

Calculate exact concentrations of each component in stock standard solution as shown below:

**Stock standard A **use concentration as is.

**Stock standard B **concentrations will require multiplication by an additional 1/10 factor.

**(c) ***Fluorescence Detector Sensitivity Assessment: Stock Standard Selection (Stock Standard Solutions A, B): *When running this method for the first time inject the appropriate injection volume, 10 μl, of Stock Standard A and B onto the HPLC system under the conditions provided in Section G. Examine the detector response for the two concentrations provided. Choose the most concentrated stock standard solution that does not saturate the detector as the Stock Standard with which to proceed. Discard the other stock standard solution. If proceeding with stock standard B, for future analysis note that the stock standard preparation procedure can be modified by preparing a stock standard solution of 0.1 mg/mL to save one dilution step.

**(d) ***Reagent Blank: *Use extraction solvent for the blank.

**(e) ***Working standards: *Prepare Working Standard Solutions from the chosen stock standard. Add indicated amounts of appropriate working standard (100 or 1000 μg/mL) to a 10 mL volumetric flask; bring to volume with the extraction solvent. Transfers should always be made with Class A volumetric pipettes. Alternatively test tubes can be used and the remaining extraction solvent for dilution to 10 mL can be added with Class A volumetric pipettes. See an example of the working standard dilution scheme in Table 1 below.

**Table 1:** Example of Working Standard Dilution Scheme(s)

Working Standard	Use one of the stock standard solution and dilute to prepare working standards in 10 mL volumetric flasks 1000 μg/mL (Standard A); or 100 μg/mL (Standard B); [mL]	Stock Standard A Approximate Working Standard Concentration [μg/mL]	Stock Standard B Approximate Working Standard Concentration [μg/mL]
Blank	0	0	0

1	0.250	25	2.5

2	1.000	50	5.0

3	2.000	200	20.0

4	3.000	300	30.0

5	4.000	400	40.0

6	5.000	500	50.0

7	Use "as is"	1000	100.0

**(f) **Calculate the exact concentration of each component of the working standards as follows:

#### E. *Lipid Removal from Cocoa and Chocolate Samples*

**(a) **Accurately weigh approximately **2 grams **of each finely divided/grated milk chocolate sample or **1 gram **for cocoa powders/baking chocolate/dark chocolate samples into a labeled, tared 50 mL disposable centrifuge tube. Record the weight of the sample W _SAMPLE_.

**(b) **Add approx. 40 mL hexane (dispenser) and cap tightly.

**(c) **Mix until the sample is completely dispersed (check visually).

**(d) **Centrifuge for 5 minutes at 2500 × g.

**(e) **Carefully decant and dispose of the hexane phase immediately.

**(f) **Repeat defatting steps **(b) **to **(e) **one additional time.

**(g) **Remove the cap and allow the residual solvent to evaporate in an appropriate fume hood until remaining hexane has evaporated (e.g. over night). Alternately, a stream of nitrogen may be used to accelerate the drying process.

#### F. *Preparation of Test Solutions*

Continue with whole sample remaining in the centrifugation tube.

**(a) **Add 2 glass beads to the centrifuge tube containing the dried, defatted sample.

**(b) **Add 9 mL of extraction solvent (dispenser) and vigorously shake the sample to break centrifugation pellet. Sample does not need to be completely suspended yet. Shake headlong, if necessary gently tap several times.

**(c) **Place in an ultrasonic bath at 40°C for 15 minutes in total. After 5-10 minutes of sonication, remove sample from bath and handshake again until sample is completely suspended (check visually). Alternately, vortex sample.

**(d) **Remove the sample from the ultrasonic bath, centrifuge at 2500 × g for 5 minutes.

**(e) **Carefully and slowly decant the liquid portion into a 20 mL Class A volumetric flask (wide neck, if possible).

**(f) **Repeat the extraction steps **(b) **to **(d) **one additional time. Decant the liquid from the second extraction into the same 20 mL volumetric flask.

**(g) **Bring to volume with extraction solvent.

**(h) **Assemble a Syringe and Syringe Filter. Filter approximately 1 mL of sample into a HPLC Vial.

**(i) **Analyze by HPLC as described in Section G.

#### G. *Chromatography*

**(a) ***Injection volume: *10 μL

**(b) ***Flow rate: *0.65 mL/min for 3 mm i.d. column; Alter flow rate to maintain linear flow for other column dimensions.

**(c) ***Detection: *Fluorescence with excitation at 280 nm and emission at 315 nm

**(d) ***Column temperature: *40°C

**(e) ***Gradient Elution: See *Table 2* for example gradient conditions*. HPLC columns differ in their selectivity for these compounds. Gradient conditions should be altered as needed to achieve resolution of (±)-catechin and (±)-epicatechin from interfering peaks.

**Table 2:** HPLC Gradient Example

Time [Minutes]	%B
0	5

30	30

31	80

35	80

36	5

40 (End)	5

**(f) ***Concentration of analytes in sample extract: *Check if concentration of analytes in sample extract lie within their calibration ranges. If necessary, dilute and re-run extraction solution.

**(g) ***Check sample: *Check sample by re-runing mid point calibration curve in middle and at end of sample sequence. Calculate the mean, standard deviation and coefficient of variation (%CV) of the peak areas.

#### H. *Calculations*

Integrate peak area for quantitation. If peak areas of analytes in sample extracts are above calibration curve, dilute sample extract solution with extraction solvent accordingly. If peak areas of analytes in sample extracts are below calibration curve, repeat sections E and F and increase sample weight accordingly.

Construct standard curves, plotting calibration standard concentration of each standard against the area of the standard peak, using linear regression. Calculate the analytes (±)-catechin and (±)-epicatechin in the original sample as follows:

Analyte in sample [μg/g] = assay concentration of analyte [μg/mL] * × [mL]/W_sample_[g]

W_Sample _[g] = initial sample weight from section **E (a)**

× [mL] = 20 mL (volume of extraction solution in volumetric flask; section **F)**

#### I. *HPLC System and Column Performance Criteria Qualification*

An HPLC column which fully resolves the analytes of interest may be used for the method. Gradient slope, flow rates and injection volumes may be altered as appropriate to accommodate columns of differing dimensions.

#### J. *System Suitability*

System suitability is a required procedure to ensure the HPLC system is working correctly. The following suitability tests are recommended to ensure correct system operation prior to initial use:

*Repeatability and carry-over: *Before running any test solutions, demonstrate the repeatability and lack of carryover of the HPLC system as follows:

**(a) ***System Artifacts: *As the first two injections of the day, analyze the blank standard twice in succession. Inspect the two chromatograms for artifact peaks from the HPLC system. Artifacts in the first chromatogram, absent in the second, indicate a buildup of impurities on the system. Artifacts present in both runs indicate impurities expected in every run. If the first chromatogram shows artifact peaks but the second chromatogram does not, inject a blank solution as the first sample in every analytical set. The presence of artifact peaks indicates impurities in the HPLC solvents, the needle wash system, or carryover in the injection system. These problems, if present, should be corrected.

**(b) ***Carryover: *Inject Standard 5 and then the blank. Carefully examine the blank injection for carryover peaks. Calculate the carryover of any peaks seen in the blanks as a percentage of the concentration found in standard 5. Carryover of standard 5 to the blank injection should be less than 0.1%.

**(c) ***Linearity of the standard curve: *Analyze each of the 5 standards, and construct a standard curve. The R^2 ^of each standard curve should be greater than 0.9990. If this linearity is not achieved, prepare fresh standards.

**(d) ***System precision: *Analyze five replicate analyses of standard number 3. Calculate the concentration of each analyte and calculate the mean, standard deviation and percent coefficient of variation (%CV) of the results. The %CV for all peaks should be ideally less than 2%.

#### K. *Samples*

Samples for analysis were prepared by the NCA Study Director and submitted as blind samples to five participating laboratories. Samples consisted of cocoa, dark chocolate, milk chocolate and NIST SRM [2384] Baking Chocolate having certified values for (±)-catechin and (±)-epicatechin. One dark chocolate samples was used as a blind duplicate to assess method repeatability.

#### L. *Quantification*

Quantification was performed using the external calibration method as described in the method with all laboratories reporting regression coefficients in excess of 0.99 with the labs equally divided whether calibration was forced through zero.

## Results and Discussion

All results were submitted to the Study Director using the form that was provided with the samples and with all data statistically evaluated. Samples were run in duplicate or triplicate. Furthermore each data set was evaluated using the Q-test to test for outliers with some data being eliminated. The results can be seen in Table 3.

**Table 3:** Flavan-3-ol Quantification

(±)-Catechin Results μg/g			
	**Avg**	**SD**	**%CV**

Cocoa 1	1681	153	9

Cocoa 2	456	49	11

Milk Chocolate 1	< 25	N/A	N/A

Milk Chocolate 2	72	9	12

Dark Chocolate 1	214	15	7

Dark Chocolate 2	246	35	15

NIST	241	24	10

**(±)-Epicatechin Results μg/g**			

	Avg	SD	%CV

Cocoa 1	3312	412	12

Cocoa 2	254	27	11

Milk Chocolate 1	35	9	24

Milk Chocolate 2	179	12	7

Dark Chocolate 1	263	18	7

Dark Chocolate 2	262	20	8

NIST	1137	68	6

N/A* These results were eliminated due to designation as outliers by the Q-test for outliers [16].

**Figure 1:**
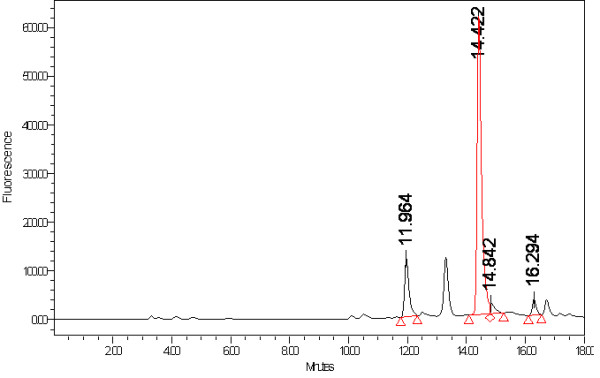
**Chromatogram of Dark Chocolate Extract Analyzed Under Method Parameters**. Peak at 11.964 is (±)-catechin while peak at 14.422 is (±)-epicatechin.

Figure 1 is a sample chromatogram provided from one laboratory of a dark chocolate extract. Instrumental conditions are as described in the methods section.

Each laboratory also provided information about LOD (Limit of Detection) which was in pure solvent in the 40-50 ng/mL. The LOQ (Limit of Quantitation) ranged from 1- 2 μg/g Repeat injections of standards were also accomplished with %CVs reported in the 1-4% range.

The NIST (National Institute of Standard Technology) reference values for (±)-catechin and (±)-epicatechin in SRM (Standard Reference Material) 2384 are 245 +/- 51 (μg/g) and 1220 +/- 22 (μg/g) respectively with the data from this study indicating value of 254 +/- 23.5 (μg/kg) and 1137 +/- 68 (μg/kg) which are within the acceptable range of determinations established by NIST. While recovery studies have become a default method to assess method accuracy according to Swartz and Krull, the analysis of an established SRM is by itself a generally accepted method of validation [17]. Additionally, guidance from AOAC on methods validation indicates that spiking is not a desirable method to assess method accuracy as spiking solutions tend to be easily extractable hence the choice of the NIST standard to evaluate the method.

The sample labeled Milk Chocolate 1 is an example for a product containing very low amounts of the target analytes. With the analyte concentration in the sample extract at their lower limit of quantification and the chromatographic performance negatively affected by co-extracted matrix compounds the applied method operates at its limit. Hence the sample was not included in the statistical evaluation of the method. That being said, no issues related to complexation of polyphenols with milk reported by some researchers were seen [18].

The %CV ranged from 7-15% in this study. Laboratories used a column that satisfied the requirements of U.S. Pharmacopeia, previously described in methodology section [19]. The method was reviewed and compared with recommendations of Swartz and Krull. Finally, while the data in Table 3 may seem excessive to the casual observer, it is well within the parameters established by AOAC for another complex analyte [20].

The literature reports on the use of numerous solvents for the extraction of flavan-3-ols including mixtures of methanol, acetone, water and acid therefore the solvent combination used is within established parameters [21,22]. Furthermore, a variety of HPLC detector types have been used including UV, Diode Array, Mass Spec and fluorescence [23-27]. The choice of fluorescence detection is within established analytical parameters for this determination as it offers selectivity and sensitivity for these compounds with the identity of the peaks being established by the use of authentic standards.

## Conclusion

The data from these studies indicate the proposed chocolate and cocoa method is suitable as an HPLC method for the determination of flavanol monomers, (±)-catechin and (±)-epicatechin in chocolate and cocoa. The method is the first such method developed by an industry group such as NCA for this purpose.

## Abbreviations

HPLC: High-Performance Liquid Chromatography; ISO: International Organization for Standardization; mL: Milliliter;°C: Degrees Celsius; nm: Nanometer; mm: Millimeter; μm: micrometer; PVDF: Polyvinylidene Fluoride; PTFE: Polytertrafluoroethylene; L: Liter; mg: Milligram; g: Gram; Min: Minutes; μg: Microgram; %: Percent; NIST: National Institute of Standards and Technology; SRM: Standard Reference Method; LOD: Limit of Detection; LOQ: Limit of Quantification; AOAC: Association of Official Analytical Chemists; UV -Ultraviolet

## Competing interests

The authors declare that they have no competing interests.

## Authors' contributions

AB selected standard and sample preparation methods, chromatography parameters and suitable commercial samples. LS distributed samples to participating laboratories, collected and analyzed data and prepared the manuscript. All authors read and approved the final manuscript.
